# An overview of microneedle applications, materials, and fabrication methods

**DOI:** 10.3762/bjnano.12.77

**Published:** 2021-09-13

**Authors:** Zahra Faraji Rad, Philip D Prewett, Graham J Davies

**Affiliations:** 1 School of Mechanical and Electrical Engineering, University of Southern Queensland, Springfield Central, QLD 4300, Australia; 2 Department of Mechanical Engineering, University of Birmingham, Birmingham B15 2TT, United Kingdom; 3 Oxacus Ltd, Dorchester-on-Thames, OX10 7HN, United Kingdom; 4 Faculty of Engineering, UNSW Australia, NSW 2052, Australia; 5 College of Engineering & Physical Sciences, School of Engineering, University of Birmingham, Birmingham, B15 2TT, United Kingdom

**Keywords:** drug delivery, microelectromechanical systems (MEMS), microfabrication, microneedles, point-of-care diagnostics

## Abstract

Microneedle-based microdevices promise to expand the scope for delivery of vaccines and therapeutic agents through the skin and withdrawing biofluids for point-of-care diagnostics – so-called theranostics. Unskilled and painless applications of microneedle patches for blood collection or drug delivery are two of the advantages of microneedle arrays over hypodermic needles. Developing the necessary microneedle fabrication processes has the potential to dramatically impact the health care delivery system by changing the landscape of fluid sampling and subcutaneous drug delivery. Microneedle designs which range from sub-micron to millimetre feature sizes are fabricated using the tools of the microelectronics industry from metals, silicon, and polymers. Various types of subtractive and additive manufacturing processes have been used to manufacture microneedles, but the development of microneedle-based systems using conventional subtractive methods has been constrained by the limitations and high cost of microfabrication technology. Additive manufacturing processes such as 3D printing and two-photon polymerization fabrication are promising transformative technologies developed in recent years. The present article provides an overview of microneedle systems applications, designs, material selection, and manufacturing methods.

## Introduction

The concept of microneedle structures to penetrate painlessly the outermost layer of the skin, the stratum corneum (SC), was first introduced in 1976 [1]. However, the lack of microfabrication technologies delayed the experimental research of the concept until the 1990s when developments in microfabrication tools facilitated the manufacturing of microstructures and microelectromechanical systems (MEMS) and provided a platform for microfabrication of compact miniaturized medical devices for human health screening, monitoring, and diagnostic purposes. Microneedles are microstructures that are sharp and robust enough for skin penetration, made using MEMS technology. The application of microneedle patches to the skin produces microsized pathways for transporting molecules, including biomedical antigens and cells. There have been many studies of microneedles for applications such as drawing blood and interstitial fluid (ISF) or delivering low and high molecular weight biotherapeutics, drugs, and vaccines through the skin. A wide range of microneedle structure, design, geometry, and microneedle array densities is manufactured using different rapid prototyping and microfabrication technologies such as deep reactive ion etching (DRIE) [2], lithography [3], hot embossing [4], and micromoulding [5]. In addition to microneedles for skin penetration, these microstructures have also been used in other sites of the body including the delivery of bioactive drugs into the eyes [6] and the insertion of molecules into cells using nanoneedles [7–8]. The present article reviews applications, materials, and fabrication of microneedles.

## Review

### The advantages of microneedles

#### Drug and vaccine delivery

Microneedle devices have potential advantages over traditional hypodermic needles for drug and vaccine delivery. Microneedles are less invasive, with dimensions designed to avoid stimulating nerves and causing discomfort to the patient. Human skin penetration experiments have demonstrated the reduced pain associated with microneedle penetration and the effect has been quantified using the visual analogue scale (VAS), showing an approximately 90% reduction in pain for a microneedle penetrating 480 µm, compared with a conventional hypodermic needle which penetrates several millimetres into the skin [9–10]. The pain was marginally greater for a 700 µm microneedle, but even a microneedle over 1 mm long produced a pain reduction of over 60%.

Moreover, pharmacokinetic profiles of drug and vaccine delivery by conventional hypodermic needles are not ideal and accidental needle injuries or deliberate misuse or reuse are unfortunately commonplace. In contrast, microneedle devices in the form of cheap disposable patches, have the potential to be administered without clinical expertise, or even self-applied, to improve the pharmacokinetic profile of therapeutic component delivery, remove the risk of needle stick injury, and reduce “sharps” and other biohazardous waste. For example, disposable microneedle patches could reduce the transmission of HIV by promoting the growth of self-administration of tests and treatments, particularly in transitional and developing countries. Although oral delivery of drugs may overcome some of these problems, many drugs cannot be absorbed orally because of degradation in the liver and gastrointestinal tract [11], so that intravenous, intramuscular, or subcutaneous injection of therapeutic agents is still a very common practise in all healthcare settings. Unlike conventional immunisation, which is typically accomplished by high vaccine dose, microneedle patch delivery utilizes a significantly lower dose of vaccine by targeting the rich immune system of the skin to give greater immune response and more efficient use of the antigen [12]. Such enhanced responses are a likely requirement of future uptake of microneedle delivery systems due to their small active area. Fortunately, there are encouraging data from recent coronavirus-related research [13] and from cancer research (25% enhanced absorption of the protein cancer drug Avastin^TM^ compared with conventional hypodermic delivery) [14]. Incorporation of drug-loaded nanoparticles in dissolving microneedles also shows promise for dose concentration, for example using the antimicrobial carvacrol (CAR) [15]. Doses sustained over time could be achieved using slowly dissolving structures or through stepwise bolus using multiple patches. Incorporation of hydrogel reservoirs in microneedle patches is a plausible alternative to conventional drug pump delivery systems and there has been some relevant work on hydrogel-forming polymer microneedles [16–18].

Microneedle patch technology has the potential to overcome the challenges involved in mass vaccination against COVID-19 across the world and has already shown promising achievements in delivering lyophilised or liquid formulation-based vaccines and macromolecules including influenza vaccines and insulin [19–21]. Research showed effective delivery of solid-state influenza vaccine into mice skin by microneedles [22], and delivery of macromolecular drugs to deep skin tissues of rats by a minimally invasive system consisting of microneedles and skin electroporation [23]. In particular, microneedles facilitate transdermal delivery of water-soluble and high molecular weight drugs.

**Figure 1:**
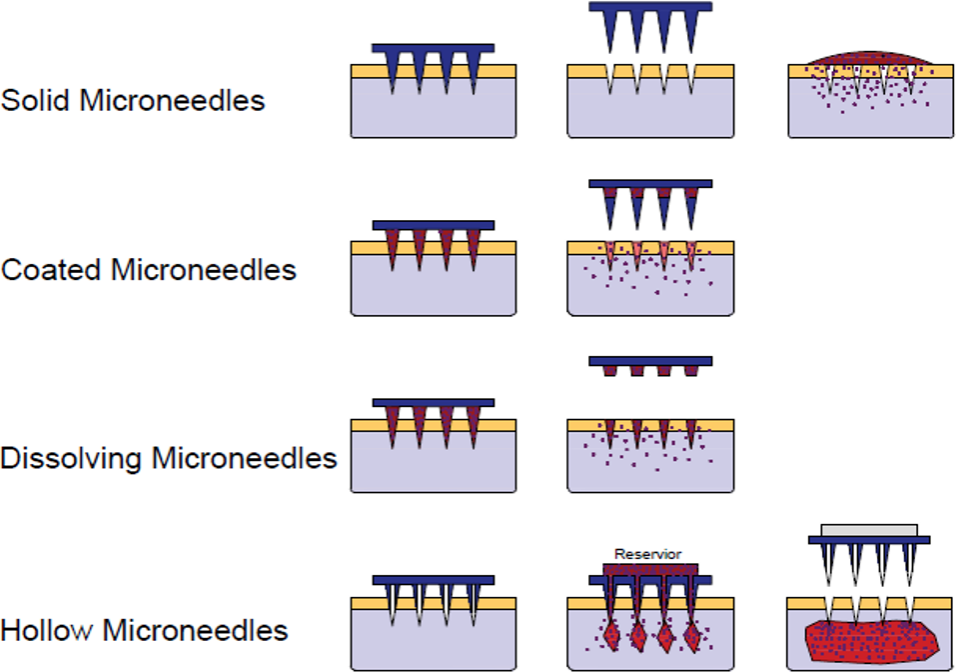
Schematic illustration of methods of microneedle application to the skin for drug delivery purposes.

Several microneedle designs enable drug delivery into the skin. Hollow or side-open microneedles allow pressure-driven or diffusion of drugs [24]. Solid microneedles may be pre-coated with a drug before insertion, or can be used to puncture the skin before or after the application of a drug to the skin surface. Drug-coated microneedles will release the drug from their surface into the skin once the coating is hydrated by body fluids (Figure 1). This method of delivery is dependent on the small surface area of the individual microneedles and is therefore more suitable for vaccination and delivery of the most potent drugs [25]. The microneedles may be made from soluble materials such as a combination of polyvinyl acetate and sucrose which dissolve to release the incorporated drug. A wide range of molecules including the anthrax vaccine [26], aminolevulinic acid [27], calcein [28], erythropoietin [29], bovine serum albumin [30–31], ovalbumin [32], insulin [33–34], and plasmid DNA [35] have been transdermally delivered using microneedles of various designs, aimed at a range of medical applications. However, the delivery of therapeutic agents by dissolving or coated microneedles has encountered problems, such as heating of carbohydrates and polymers, which can cause drug breakdown during moulding of microneedles at raised temperatures [36]. Research to overcome these issues has included fabrication of microneedles from aqueous mixtures of amylopectin and carboxymethylcellulose, rather than molten polymers, which helps to preserve the stability of the incorporated drug [37]. Accurate coating of microneedles is another challenge, but degradable or coated microneedles potentially allow lower controlled volumes of therapeutic agents to be delivered compared to hollow microneedles, which are also more prone to becoming clogged by microscopic debris during insertion [38].

The rate of drug diffusion from the microneedle surface into the patient will depend on the hydrophilicity and size of drug molecules or vaccines, and the depth of microneedle penetration is particularly important in many applications such as delivery of pharmacological agents where clinical imperatives demand a rapid onset of their action, as in emergency settings.

Means of storing and delivering useful dosages of drugs and vaccines is a key issue, since the active area of microneedles in a patch device is limited by the force which must be applied to achieve skin penetration. This is a clear limitation of the dissolving microneedle approach [37]. A reservoir, separate from the microneedles, for example using porous silicon, is one possible solution [39–41]. However, a hydrogel reservoir which could be much larger than the microneedle array seems a better option, since it can swell to achieve greater load which can be released under finger pressure in combination with microfluidic channelling of the drug load to the active area [42]. It is likely that some increase in drug concentrations will be necessary, while applying multiple patches over time would mimic catheter infusions.

#### Point-of-care diagnostics

In addition to drug delivery, microneedles may be used for drawing blood or interstitial fluid for point-of-care clinical diagnostics. As sensitive, rapid, early diagnosis and treatment of diseases are often critical, microneedle patches may soon play a vital role in point-of-care theranostics. By integrating microneedles with microfluidic chips capable of in situ measurements of human metabolic parameters (such as blood glucose in diabetic patients), diseases may be diagnosed by the observation of clinical symptoms informed immediately by micropatch biochemical analyses. Traditionally, testing is performed in laboratories by medically trained scientists and technicians, often requiring large, expensive equipment. Point-of-care diagnostics performed by a paramedic or other first responder, or in a community clinic setting, will de-skill the tests, greatly reduce costs, and accelerate the route to treatment with particular advantages in life-threatening emergencies such as heart attacks. In this context, microneedle patches have the potential to save lives [43–44].

Several studies have shown that microneedles are capable of withdrawing blood and ISF by capillary action alone without the need for negative pressure (suction) [44–45].

The application of microneedle-based devices is not limited to biological fluid extraction or vaccine and drug delivery. For example, silicon microneedles with heights of 320 and 400 µm were fabricated for in vivo human gene therapy [35]. Microneedles have also been used for treatment of hypertension in hemodynamic and cardiovascular disorders in a study which integrated hollow silicon microneedles with a reservoir unit, a piezoelectric actuator system, and a flow sensor for real-time measurements of fluid dynamics [46]. In other research, much smaller microneedles – just 8 μm in height and 1 μm in diameter – were fabricated by DRIE for simultaneous injection of particles into cells [47]. Microneedles have also been used for diagnosing allergy [48], for cosmetic applications [49], dissolvable delivery of drugs into neural tissue [50], and as microelectrode arrays for neural probes [51]. They have also been used for the detection of different skin diseases including cancer, via electrical impedance measurements [52].

### Microneedle structure design

Several factors should be considered when designing microneedles for skin penetration: (1) geometric features, such as length, diameter, tip size, and shape, (2) physical form: solid, hollow, side-opened, conical, bevelled tip, (3) material selection, (4) fabrication feasibility, (5) application, (6) layout of the arrays, (7) density, (8) total number of microneedles, and (9) surface layer state (e.g., hydrophobicity). In addition, microneedles are defined according to their array density, length or height, and shaft and tip shape. Other parameters including fluid flow rates, biocompatibility, penetration force, fragility, relative simplicity, and cost of fabrication are all key design considerations. The final design will depend on the limitations of the fabrication method and the mechanical properties, physical, and chemical stability of the material.

Most microneedles to date have been fabricated with heights below 1 mm, yet sufficient to access ISF or capillary blood or to deliver therapeutic agents. Some obstacles to insertion of microneedles into the skin are the presence of dermatoglyphics (small wrinkles) and hair, while the passage of cells through hollow microneedles depends on their internal lumen diameter, which must be large enough to ensure the flow of microscopic entities, including larger objects such as white blood cells (leukocytes), which are tens of microns in diameter. Other parameters determining flow in a microchannel include blood viscosity, contact angle, hydrodynamic diameter, and driving forces such as surface tension. In addition, due to the elastic nature of the skin and its irregular surface, varying from person to person, and with age and position on the body, the efficient penetration of microneedles to the desired depth, without fracture and with high accuracy, may require an applicator to facilitate skin penetration in a controlled and reproducible manner.

**Figure 2:**
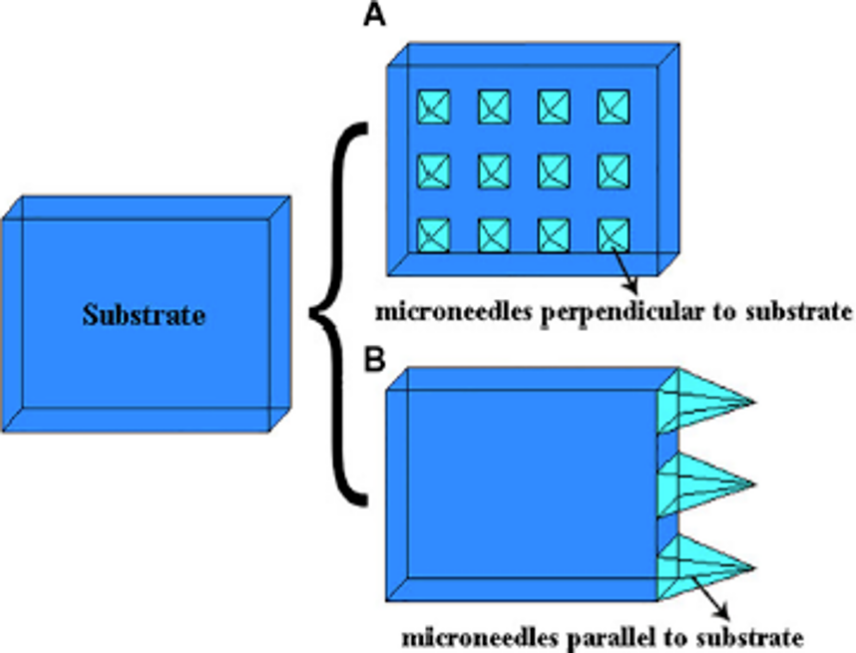
Schematic showing in-plane and out-of-plane microneedle arrays [53]. Figure 2 was reproduced from [53] (© 2019 X. He et al., published by SAGE, distributed under the terms of Creative Commons Attribution-NonCommercial 4.0 License (http://www.creativecommons.org/licenses/by-nc/4.0/). This content is not subject to CC BY 4.0.).

The microneedle design will vary depending on the application and fabrication method used (e.g., solid, hollow, open groove) but all microneedles can be classified as in-plane or out-of-plane (see Figure 2) [53]. In-plane microneedles have the longitudinal axis of the shaft parallel to the surface of the substrate while out-of-plane microneedles have their longitudinal axis perpendicular to the substrate surface. Fabrication of two-dimensional arrays of in-plane microneedles is very difficult, but it is easier to integrate in-plane microneedles with micropumps, sensors, microfluid chips, and electronic circuitry. On the other hand, it is significantly more convenient to fabricate arrays of out-of-plane microneedles in high-density two-dimensional arrays, although the resulting microneedles are restricted to lower aspect ratio and shorter height compared to in-plane microneedles if traditional microfabrication methods, such as wet and dry etching, are used.

Hollow microneedles contain a lumen or internal channel for pressure-driven fluid communication through the microneedle and the skin [12,54]. The fluid can be a drug, vaccine, blood, or ISF. This design enables the transporting of drug solutions and vaccines rather than their dehydrated form as is the case for dissolving microneedles or the competing needle-free methods, including powder jet delivery [55]. Solid microneedles are simpler to manufacture than hollow microneedles, so most preliminary studies were performed on solid versions. For solid microneedles, the vaccines or drugs are either coated on the microneedle surface or applied to the skin after micropores have been formed by the insertion of microneedles [12]. Therapeutics pre-coated on solid microneedles may dissolve off them after insertion into the skin. Gas-jet dry coating [56], liquid methods including repeated immersion and dip-in coating [57–58], and spray coating [56,59] are some of the techniques used for coating microneedle arrays with drugs and vaccines (mainly water soluble). Another approach for drug delivery using solid microneedles is to fabricate them entirely from biodegradable or water-soluble dissolving polymers. Drugs are encapsulated into the microneedle body and released as the microneedles dissolve [34,37]. The main limitation of dissolvable microneedles is the limited choice of drugs that can be encapsulated into dissolvable polymer or polymer–sugar combinations [34,36], and the short time and low volume of drug delivery. Dissolvable polymer microneedles of soluble poly(lactic-*co*-glycolic acid) (PLGA) and PLGA–polyvinylpyrrolidone (PLGA–PVP) layered combinations have been used to provide controlled drug delivery of bovine serum albumin (BSA), rather than instantaneous release [60].

There are only a few published studies demonstrating the fabrication of microneedles with an open channel design [2,5]. Like hollow microneedles, open channel microneedles provide constrained flow of fluids which can be used for both extracting blood and ISF and for delivering drugs. They are designed to be less vulnerable to blocking by fragments of dermal tissue. They can be made using micromoulding techniques, including hot embossing, which cannot be implemented for hollow microneedles because of residual debris in the lumen [5].

### Microneedle materials, manufacturing methods and uses

#### Overview of manufacturing methods

Arguably, the most challenging problem for the field has been the availability of low-cost manufacturing methods to unlock the clinical implementation of microneedles; the most important materials and manufacturing methods are presented below.

Microneedles have been fabricated from various materials starting with silicon, in different shapes and sizes for a wide range of applications. Metals such as titanium [61–62], stainless steel [58,63–64], silicon [65–67], ceramics [68–69], biodegradable polymers such as polylactic acid (PLA) [70], PLGA [71], and polyglycolic acid (PGA) [72], and non-degradable polymers such as photolithographic epoxy [73] have all been used.

Microneedles were first made from silicon as the microelectronics industry provided tools for manufacturing integrated circuits that could be adapted to microneedle fabrication [74] and silicon is still the most common microneedle material. Although this technology has aroused widespread interest and provides potential for mass production, the manufacturing technology requires complex multistep processes using expensive equipment located in dedicated cleanroom facilities originally intended for planar integrated circuit designs and adapted for microelectromechanical systems. Alternative manufacturing processes, such as 3D printing and two-photon polymerization (TPP), are promising new transformative technologies developed in recent years. These additive manufacturing methods use layer-by-layer processing to create 3D structures. Unlike other microfabrication methods developed for microneedles, these rapid prototyping methods do not require expensive cleanroom facilities, and complex geometries can be realised in a shorter time and with less technical expertise. This is a major advantage for fabrication of microneedle patch arrays requiring integration of microfluidic elements for point-of-care diagnostics or drug delivery. Recent commercialization of TPP microtechnology by companies such as Nanoscribe GmbH (Germany) has enabled precise and flexible fabrication with submicron resolution [5].

Other manufacturing processes for microneedle fabrication include injection moulding [61], wet chemical etching [75], reactive ion etching [2,76], hot embossing [4–5], laser drilling [77], lithography plus electroforming [78–79], drawing lithography [80–81], two-photon polymerization [5,82], and 3D printing [83–84]. To date, DRIE of silicon; micromoulding; photolithography; and Lithographie, Galvanoformung, Abformung or lithography, electroplating, moulding (LIGA), using deep X-ray lithography, are the most extensively used manufacturing technologies for microneedle fabrication, although fabrication of longer microneedles (>400 μm) is difficult with some of these methods, notably DRIE. Drawing lithography has been used for high aspect ratio microneedles of heights 1600, 1200, and 600 μm [80–81]. This technique involves spin coating of a viscoelastic thermosetting polymer such as SU-8 epoxy resin, followed by thermal curing and controlled drawing of the material in liquid form. It has so far been limited to low density arrays with relatively large spacing between adjacent microneedles (>900 μm).

Faraji Rad et al. made tall polymer microneedle arrays with complex design using TPP and micromoulding [5]. Two-photon polymerization enables fabrication of almost any microstructure directly from the CAD design file.

We now consider in more detail the most important microneedle materials and their fabrication and uses.

#### Fabrication and use of silicon microneedles

Silicon microneedles have, to date, been the most common type, using microfabrication methods with complex multistep processes and expensive tools developed for the microelectronics industry [74,85], as introduced already. Subtractive technologies, in the form of wet and dry etching, are most frequently used. In wet etching, a single crystal silicon wafer is immersed in baths of various chemical etchants for either isotropic etch (the etch rate is the same in all directions) or anisotropic etch in which the etch rate differs for different crystal planes [74,85]. Anisotropic etching cannot form cylindrical microneedles, since the etch exposes selective crystal planes which produce instead faceted pyramid microprotrusions. In both isotropic and anisotropic cases, a hard mask layer of silicon nitride is first patterned using optical lithography. Isotropic etching uses a highly corrosive HNA solution containing hydrofluoric, nitric, and acetic acids. This produces undesirable undercutting of the etch mask which must be compensated for in the pattern design. Anisotropic etching uses either EDP (ethylenediamine pyrocatechol), hydrazine-based solutions or, most commonly, potassium hydroxide solution.

Deep reactive ion etching of silicon is an increasingly common process, performed in a low-pressure chamber where a plasma of reactive ions is formed on the material surface. Etching can be either isotropic, at higher gas pressures, or anisotropic when lower pressures are employed. The most common silicon etchant is sulphur hexafluoride (SF_6_ + O_2_) in an inductively coupled plasma etch tool. High aspect ratio etches are achieved by multiplex switching of gas feed between SF_6_ + O_2_ (etch) and C_4_F_8_ (sidewall passivation) in the Bosch process [86–87]. Dry etching allows better control over microneedle density and geometry and the absence of crystal plane effects allows microneedles with cylindrical symmetry to be formed. On the other hand, wet etching has lower tool costs and facilitates mass production due to simultaneous parallel fabrication using several silicon wafers at once; however, it is limited to low aspect ratio structures. Fabrication of silicon microneedle profiles generally involves isotropic and/or anisotropic wet- and/or dry-etching processes on the front side of the silicon wafer. Silicon has been used to manufacture both solid and hollow microneedle designs. The addition of a lumen for hollow microneedles requires deep etching of silicon from the backside of the silicon wafer using an additional mask which must be aligned to the front side pattern – a process requiring through-wafer alignment using infrared light.

**Figure 3:**
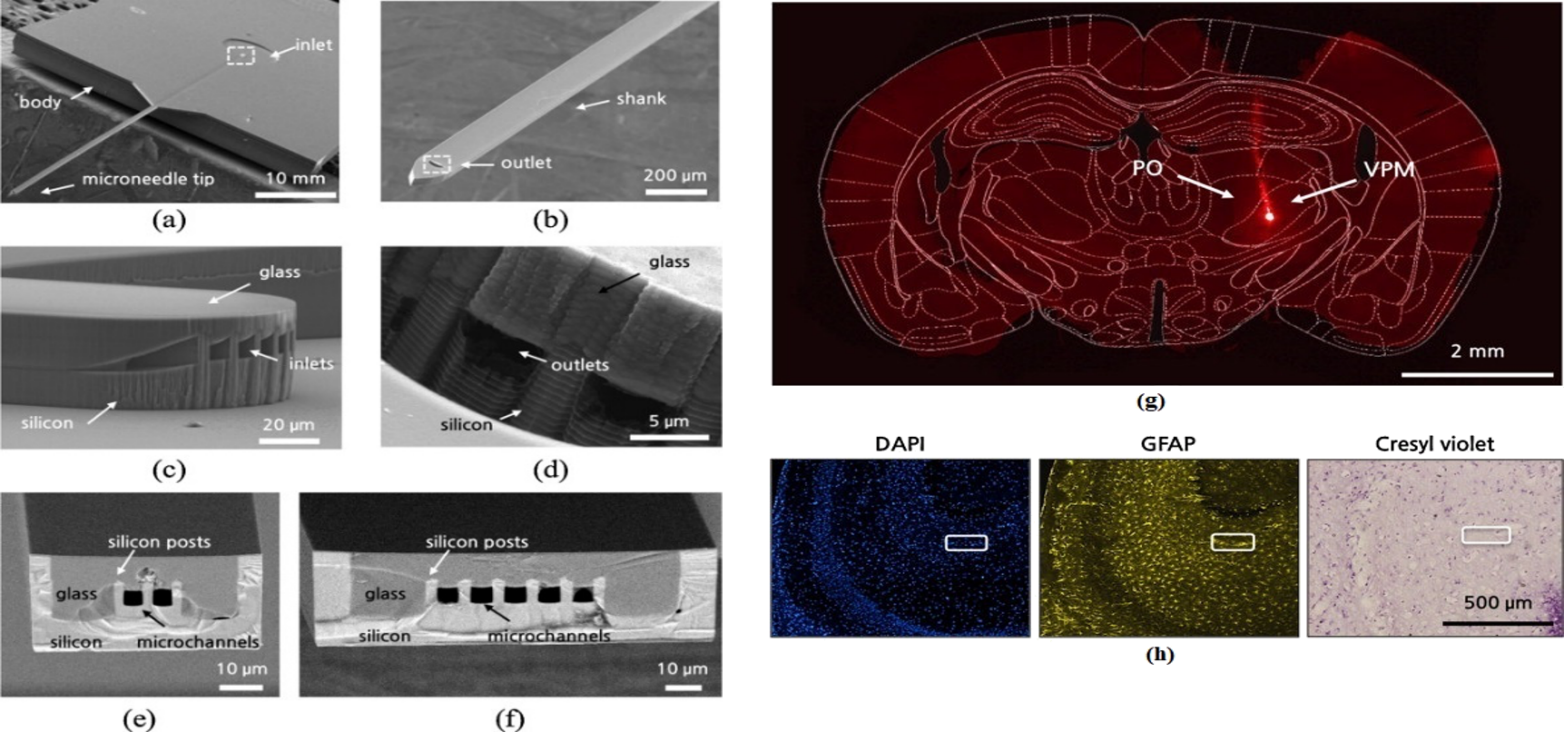
Scanning electron microscopy (SEM) image of a 5.3 mm long silicon microneedle fabricated by GCoS. (a) Overview, (b) microneedle outlet and shank, (c) inlet with microchannels, (d) outlet with microchannels, (e,f) cross-section of microchannels with two and five cavities. (g) A coronal brain cross-section micrograph with the infusion of a dye at the posterior nucleus, (h) a horizontal cross-section of brain displaying cells (Hoechst staining), astrocytes (GFAP staining), and neurons (cresyl violet staining) at the insertion location of the microneedle [66]. Figure 3a–h were reprinted from [66], Sensors and Actuators B, Chemical, vol. 209, by Lee, H. J.; Son, Y.; Kim, D.; Kim, Y. K.; Choi, N.; Yoon, E.-S.; Cho, I.-J., “A new thin silicon microneedle with an embedded microchannel for deep brain drug infusion”, pages 413–422, Copyright Elsevier (2014), with permission from Elsevier. This content is not subject to CC BY 4.0.

Other recent fabrication techniques include fixing a glass cover on silicon (GCoS) to manufacture microneedles for deep brain drug infusion. In this technique a glass wafer is anodically bonded to a silicon wafer with predefined cavities (Figure 3) [66]. Deng et al. produced a solid pyramidal silicon microneedle array for in vivo delivery of the cholesterol-modified housekeeping gene siRNA to mice ear skin. The microneedles were 200 μm in height with a tip radius of 1 μm formed by isotropic etching with static and dynamic etching steps [88]. Bolton et al. produced tall hollow silicon microneedles by three-step DRIE process [89]. Hamzah et el. fabricated sharp solid silicon microneedles, via wet etching with HNA, with approximately 160 μm height and a base diameter of about 111 μm for transdermal drug delivery [90]. Another work has shown fabrication of more complex structures, such as tapered hollow microneedles, by DRIE. In this study, the microneedle channels were first etched from the silicon wafer backside before the microneedles were formed by consecutive switching between isotropic and anisotropic etching from the front side of the wafer. Microneedles with heights between 310 and 400 μm with sharp tips were obtained [91].

Silicon microneedles are brittle and may break during insertion into the skin, which could result in foreign body reactions such as abscess formation or granulomas. The enzyme systems of the human body do not break down bulk silicon, so silicon fragments may remain in tissue for life, causing scarring and fibrosis. However, porous silicon is different, with its bioactive ability to bond to living tissue and for its biodegradable and biocompatible nature. It was first shown in 1995 that, by introducing porosity into silicon, the material behaviour can change to provide a bioactive and even resorbable material [92]. Unlike bulk silicon, in alkalescent media (pH ≈ 7.5), porous silicon is broken down by hydrolysis in living organisms. In addition, highly porous silicon (>50%) with nanoscale pore channels in the range of 5–25 nm can be used as biocompatible containers for loading and release of drugs [39]. The porosity of the porous silicon particle determines the effectiveness of the drug loading with bigger pore sizes being used to accommodate large organic molecules [39,92]. In physiological environments, porous silicon microneedles are capable of biodegradation at a rate of dissolution depending on the chemical nature of their initial surface, the acidity of the solution, and the porosity and morphology of the particles [39–41,92]. Administration of a drug by porous silicon near the targeted organ makes it possible to achieve a therapeutic concentration in the affected area with considerably reduced collateral drug toxicity in other tissues and organs [40,93]. A drug can be loaded into the porous silicon by adsorption [92], with rates of release depending on interaction of the drug with the porous silicon. Oxidation, covalent binding, and electrostatic interactions can all be used to immobilise the drug on the porous silicon [94], while both hydrophilic and hydrophobic molecules can be loaded on porous silicon structures [40–41], for example arrays with biodegradable macroporous silicon tips produced using electrochemical anodization. A disadvantage is that the tips of the microneedles may break off and remain in the skin during the drug delivery process where they will be biodegraded only after 2–3 weeks [95]. In other work, hollow pyramidal silicon dioxide microneedle arrays, with heights of 150–200 μm, were made by oxidising microporous silicon produced by a combination of wet etching and electrochemistry [96].

Porous silicon microneedles may overcome the brittle properties of single crystal silicon and provide a degree of biodegradability, but their fabrication methods are relatively complex and involve the use of toxic and corrosive chemicals like HF. In addition, the mechanical properties of materials, including Young’s modulus, significantly degrade with increasing porosity. (Note: to our knowledge there is no quantitative data available for the compressive and tensile strength values of porous silicon as a function of porosity [97]).

Considering the challenges involved in fabricating silicon microneedles, such as complex multistep fabrication processes and the high cost, requiring expensive cleanroom tools, it is perhaps surprising that silicon still dominates the field. Moreover, the lengths of typical silicon microneedles are not sufficient for reaching blood capillaries and withdrawing blood for testing [98], while silicon microneedle manufacturing techniques are incompatible with rapid prototyping and lack the flexibility that other manufacturing methods such as 3D printing provide. These attractive alternative methods of manufacturing microneedles from polymers, instead of silicon, are considered below.

#### Fabrication and use of polymer microneedles

Polymer materials are currently receiving more interest because of biocompatibility, superior mechanical properties, low material cost, and biodegradability. The low-cost of fabrication is an additional advantage of polymer microneedles over silicon. It is increasingly clear that the favoured fabrication methods used to develop the next generation of polymer microneedle point-of-care tests and drug delivery patches will be photolithography, replica moulding, 3D printing, and micromachining.

Photolithography involves polymerization of a liquid material by chemical modification through exposure to short wavelength light. The resist is sprayed or spin coated onto a substrate surface for patterning and is exposed to light (usually ultraviolet) either through a contact mask or using a projection stepper, followed by wet development to form a resist pattern. This technique requires well-established photosensitive materials such as polymethylmethacrylate (PMMA) or SU-8 epoxy resin chemically amplified resist. The former is a positive-tone photoresist in which chemical bonds undergo scission upon exposure to the UV light, rendering the exposed regions of the pattern more soluble in the developer. For negative resists, the exposure to UV light creates bonds through crosslinking whereby the exposed areas become less soluble in the developer [74,85].

In replica-moulding or micromoulding processes, a master (hard) mould is first produced. A liquid polymer solution, like polydimethylsiloxane (PDMS), is then deposited into the master mould and solidified by raised temperature (thermosetting), cooling, or UV curing to produce crosslinking. It can then be peeled from the master mould, which can be repeatedly reused to produce more replica moulds. To reduce the adhesion of the soft replica mould, the surface of the master mould may be chemically treated to facilitate the release. The polymer microneedles are typically formed by an embossing or micromoulding processes using the replica soft moulds.

**Figure 4:**
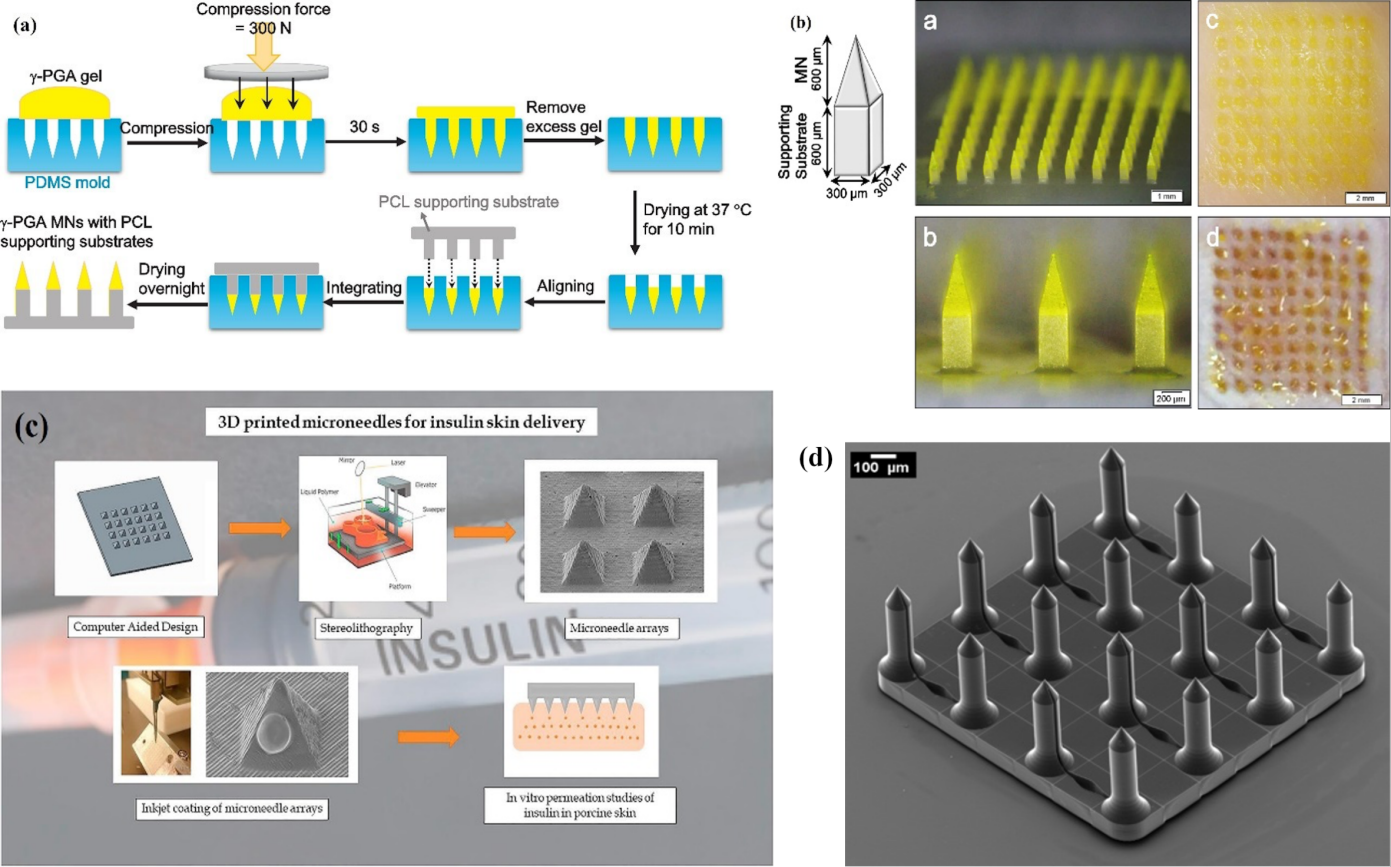
(a) A schematic representation of the manufacturing procedure for producing γ-PGA microneedles, (b) microneedles fabricated via the procedure represented in (a) as used to penetrate pig and mouse cadaver skin [72]. (c) Stereolithography (STL) used to manufacture microneedle arrays followed by coatings of insulin and sugar solution using an inkjet printer [83]. Figure 4c reprinted with permission from ref. [83]. (d) An array of 16 microneedles (2.17 mm × 2.17 mm) with side channels connected to reservoirs, fabricated by two-photon polymerization; the microneedles have 700 μm of total height, 150 μm of flange height, and 150 μm of tip height [5]. Figure 4a,b were reprinted from [72], Acta Biomaterialia, vol. 114, by Chen, M.-C.; Chen, C.-S.; Wu, Y.-W.; Yang, Y.-Y., “Poly-γ-Glutamate microneedles as transdermal immunomodulators for ameliorating atopic dermatitis-like skin lesions in Nc/Nga mice”, pages 183–192, Copyright Elsevier (2020), with permission from Elsevier. This content is not subject to CC BY 4.0. Figure 4c was reprinted from [83], International Journal of Pharmaceutics, vol. 544, by Pere, C. P. P.; Economidou, S. N.; Lall, G.; Ziraud, C.; Boateng, J. S.; Alexander, B. D.; Lamprou, D. A.; Douroumis, D., “3D printed microneedles for insulin skin delivery”, pages 425–432, Copyright Elsevier (2018), with permission from Elsevier. This content is not subject to CC BY 4.0.

Different types of polymer have been used to manufacture polymer microneedles in this category of process, including dissolving versions for drug delivery [27,37,99]. Figure 4a is a schematic of the manufacturing procedure for γ-PGA microneedles and Figure 4b shows the manufactured array [99]. In another study, photolithography was first used to create master structures from SU-8 photoresist by UV photolithography, moulding, and casting steps, forming polymer microneedles with a drug loaded onto their tips [72]. Elsewhere, transparent polymer microneedles were made in PMMA using a hot embossing process. First the master microneedle array, 250 μm high, was fabricated by a combination of isotropic and anisotropic etching using an inductively coupled plasma (ICP) etcher, then the polymer microneedles were replicated using a PDMS negative soft mould and hot embossing [100].

SU-8 photoresist has been used, not just for master moulds, but also as the final material for microneedles. Long hollow microneedles, ≈1500 μm high, were formed by exposing SU-8 polymer, on a silicon wafer, to UV light through a mask consisting of hollow circular patterns or annuli [101], followed by wet development to obtain the final microstructure.

In recent years, techniques such as 3D printing and TPP have received great interest due to their low-cost and ease of fabrication compared to multistep MEMS manufacturing processes. Figure 4c shows an example of 3D printed microneedles for insulin delivery [83].

Faraji Rad et al. fabricated open channel microneedle arrays using a combination of TPP, soft replica PDMS moulds, and soft embossing [5]. The process, which has been patented [102], is capable of producing high-fidelity high aspect ratio polymer microneedles with a variety of surface features including open microchannel grooves for fluid extraction and control. Figure 4d shows an example of microneedles in cyclo-olefin polymer (COP) material using this method [5].

#### Fabrication and use of metal microneedles

**Figure 5:**
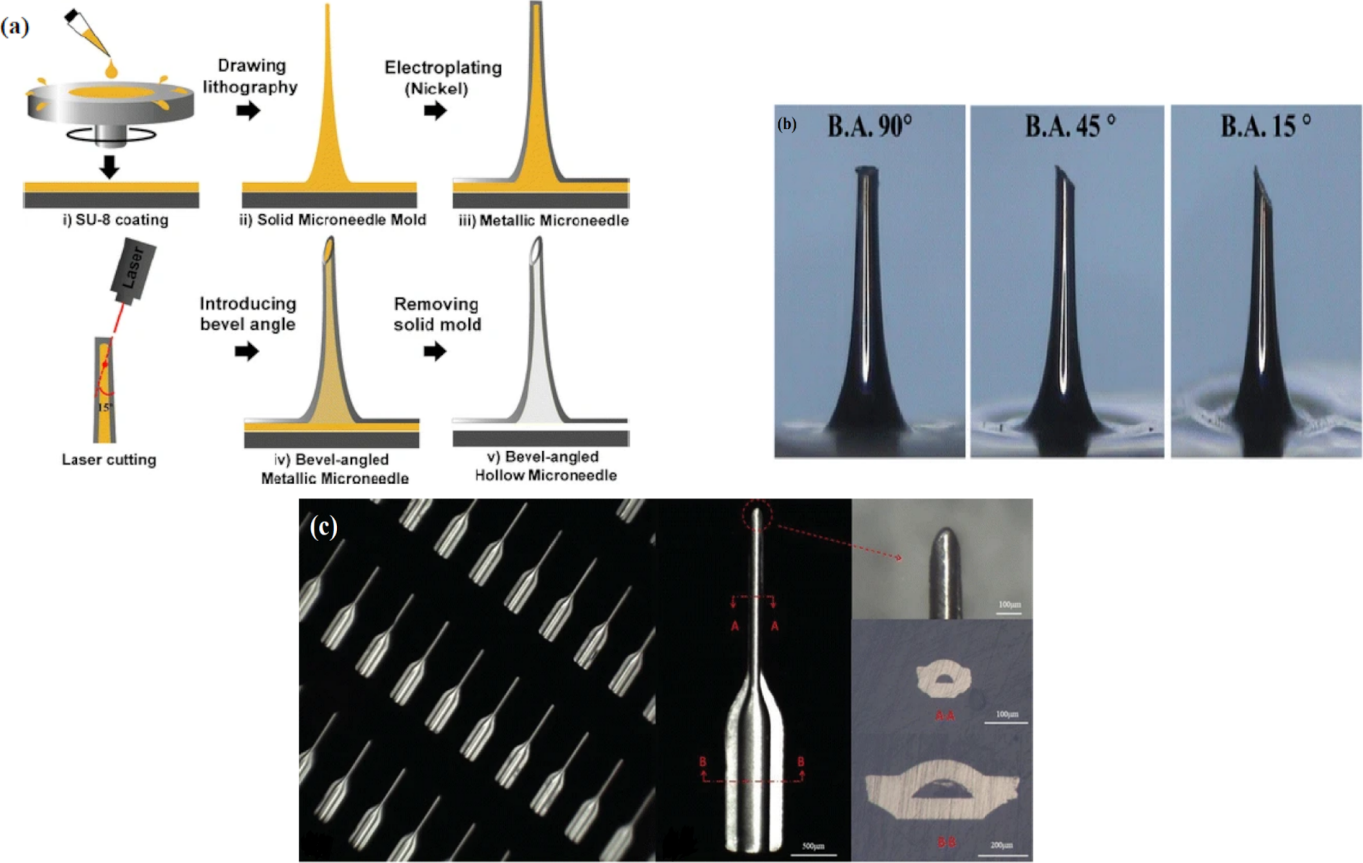
(a) A schematic illustration of the drawing lithography procedure for fabrication of nickel microneedles, (b) microneedles with 1800 μm in height, bevel angles of 90°, 45°, and 15° and an inner diameter of 60 μm for blood extraction [104]. (c) Hollow nickel microneedles with cross sectional views [105]. Figure 5a,b are from [104] and were reprinted by permission from Springer Nature from the journal Biomedical Microdevices (“An optimized hollow microneedle for minimally invasive blood extraction” by Li, C. G.; Lee, C. Y.; Lee, K.; Jung, H.), Copyright 2012 Springer Nature. This content is not subject to CC BY 4.0. Figure 5c is from [105] and was reprinted by permission from Springer Nature from the journal Biomedical Microdevices (“A minimally invasive micro sampler for quantitative sampling with an ultrahigh-aspect-ratio microneedle and a PDMS actuator” by Liu, L.; Wang, Y.; Yao, J.; Yang, C.; Ding, G.), Copyright 2016 Springer Nature. This content is not subject to CC BY 4.0.

In addition to silicon and polymers, microneedles are also fabricated from metals including nickel, titanium, stainless steel, and palladium. Metals provide good mechanical properties and biocompatibility for microneedle manufacturing compared to silicon, notwithstanding some issues of toxicity. Photochemical etching, electroplating, and laser cutting are common techniques in the fabrication of solid and hollow metallic microneedles. Studies have demonstrated the fabrication of hollow nickel microneedles by electroplating, with heights ranging from 300 to 450 μm in a 6 × 6 array. The mould was fabricated from a two-layer thick SU-8 epoxy resin master structure [103]. Microneedles with 1800 μm height, different inner diameters (40, 60, 80, and 100 μm), and a series of bevel angles were also fabricated from nickel using a combination of drawing lithography and nickel electroplating (Figure 5a, Figure 5b). The bevelled tip of the structure was achieved through laser cutting. A negative pressure of 13.45 kPa was applied to extract 20 µL of blood from the tail of a mouse [104]. In another study, nickel microneedles were fabricated using reshaped photoresist technology to form a channel inside (Figure 5c). The microneedle had a 1500 μm long shaft with a 45° angle tapered tip and a 1000 μm long pedestal. The manufacture comprised of repetitive patterning of the substrates by electroplating metal layers with multiple applications of photoresist [105].

Elsewhere, metallic microneedles were fabricated through replication of a positive mould consisting of pillars formed by photolithography in SU-8. A thick layer of nickel was electrodeposited on the mould pillars to create the microneedle array on the surface of a sacrificial layer of polymer introduced between the mould and the metal structure to facilitate chemical lift-off separation of the final array. For hollow metal microneedles, a plasma etching step was performed prior to metal deposition [106].

Unlike the ubiquitous stainless steel hypodermic needle, safety concerns with current metal microneedles may result in them having to be made from different metals. For example, the toxicity of electroplated nickel microneedles has not been adequately addressed so far in the literature. Metals are generally stronger and cheaper than polymers and silicon, but immune and inflammatory responses of biological tissues, for example to titanium and even stainless steel, present problems. From the manufacturing perspective, the fabrication of metal microneedles has complexities like electroplating and lift-off which are undesirable for mass production. Moreover, electroplating does not readily produce genuine 3D structures and the outputs of LIGA are often described as 2.5D structures. Longer microneedles, often over 1 mm, are needed in order to prevent issues associated with the porosity of electroplated metals, so that metal microneedles may not be as painless in operation as other types of microneedle [9–10].

Table 1 is an overview of the most important metal microneedle types, identifying those which are most likely to meet the challenges of mass manufacturing with selected references.

**Table 1:** Overview of key microneedle categories.

Material	Fabrication methods	Performance	Ref.

silicon	MicroFab cleanroom based. Lithography and wet etching or DRIE. Porous Si for drug loading.	Brittle, prone to fracture in application. Porous Si has better biocompatibility.	[91–92]
metal	Drawing lithograph; LIGA (hard X-ray lithography source - limited to 2D+ designs.); laser drilling; electrodeposition.	Long microneedles to avoid porous structures. Some pain in application.	[81] [77,105]
polymers	Casting, micromoulding, TPP lithography + hot embossing. Genuine 3D; Dissolving; hydrogel forming versions possible.	Short, pain-free microneedles. Biocompatible dissolving polymers (PGA, PLGA). Possibility of concentrated drug delivery using incorporated nanoparticles.	[4–5,27,99] [17–18]

Whichever type of microneedle is used, the mechanics of skin penetration provides new challenges, which are different from those of conventional hypodermic penetration. There have been several relevant studies of microneedle penetration of skin ranging from mouse tail and rabbit ear [104–105] to porcine skin [38,107] and including some data on human skin [91]. The forces required for penetration are obviously dependent on microneedle tip dimensions and skin puncture stress. Consequently, there is increased interest in ultrasharp microneedles with tips having minimum lateral dimensions below 1 µm. The penetration force linearly increases with array size and the use of controlled force mechanical inserters will almost certainly be required, building on simple spring-loaded commercial systems [108] to achieve the required forces without damaging the microneedles [109].

## Conclusion

Extensive research has recently been carried out on design, fabrication, and applications of microneedle systems. Microneedle patches, for example, could bring significant benefits from both patient and health professional perspectives due to reduced discomfort and enhanced convenience of application. In comparison to other microporation techniques such as ultrasound, thermal, electroporation, high-pressure needle-free injection, and lasers, microneedle-based systems are attracting favourable interest from both research and industry sectors. Microneedles have proved to be pain free, traversing the SC of the skin and penetrating the viable epidermis without stimulating nerve fibres. Microneedles may be integrated into biosensors, micropumps, microfluidic chips, and microelectronic devices.

The choice of manufacturing techniques for microneedles is dependent on material properties, fabrication cost, and desired height and shape of the microstructure. Hollow microneedles can actively deliver drugs into the skin, but due to their high cost and fabrication difficulties, simpler solid microneedles coated with drugs are currently attracting more attention for drug delivery. However, this design will not be suitable for extracting biological fluids. In addition, controlling the dosage of drugs for delivery will be limited by the number of microneedles on the patch and their dissolution characteristics. High cost and complexity of fabrication techniques such as DRIE of silicon and multiple processes associated with the fabrication of hollow polymer microneedles limit their potential for fluid communication in either direction through the skin. Embedding open-side channels on microneedle shafts is a novel approach, providing a passage through the skin for fluid flow and the manufacture is far easier than that for lumens in hollow microneedles.

Despite decades of research and small incremental advances in manufacturing methods, the number of licensed microneedle patch devices entering the medical devices market is still small. Most of the reported studies do not go beyond the proof-of-concept phase, and do not consider in much depth the translation aspects of their technology such as manufacturability and clinical utility. The failure to proceed beyond a proof-of-concept can be attributed to the technical limitations of the manufacturing methods most commonly applied thus far. The precision and versatility of submicron resolution 3D printing in combination with other manufacturing methods such as hot/soft embossing or injection moulding must address this gap in technology in the near future, along with high-throughput reel-to-reel processes, since the mass production of optimal microneedle patch designs and materials is a critical pathway to important clinical applications such as cheap point-of-care disposable microneedle patch diagnostics, transcutaneous drug delivery, and patch vaccination systems.
